# Tissue Plasminogen Activator as an Approved Strategy for Ischemic Stroke: A Review of tPA’s Structure, Mechanism of Action and the Novel Targeting Methods

**DOI:** 10.1007/s12031-025-02443-3

**Published:** 2025-11-17

**Authors:** Kimia Didehvar, Mehdi Haghshenas, Reyhaneh Yarmohammadi, Bardia Hajikarimloo, Roya Ghafoury

**Affiliations:** 1 Rutgers New Jersey Medical School, Newark, NJ 07103 USA; 2 Student Research Committee, School of Medicine, Iran University of Medical Sciences, Tehran, Iran; 3 Carolina University, NC Winston-Salem, USA; 4 Skin and Stem Cell Research Center, Tehran University of Medical Sciences, Tehran, Iran; 5 Department of Neurological Surgery, University of Virginia, Charlottesville, VA USA; 6 Endocrine Research Center, Institute of Endocrinology and Metabolism, University of Medical Sciences (IUMS), No. 10, Firoozeh St, Vali-asr Ave, Vali-asr Sq, Tehran, 1593716615 Iran; 7 Department of Anesthesiology, Rutgers University, 185 orange St, Newark, NJ 07103 USA

**Keywords:** Drug delivery, Ischemic stroke, Nano tPA, Neuroprotective, Neurotoxicity, Signaling pathway, Targeting, TPA

## Abstract

Tissue-type plasminogen activator (tPA) is a serine protease that contains five functional domains, and it acts through influencing different substrates, binding proteins, and receptors. Studies revealed that tPA has been observed to have both neurotrophic and neurotoxic effects. It is still unclear how these opposite functions are modulated by tPA but the degree of maturity and/or the type of neurons, structure of the tPA, origin, and amount have been suggested as effective factors. The sole FDA-approved thrombolytic medication for acute ischemic stroke is tPA, yet worries about its limits still exist. Due to tPA’s limitations, conventional thrombolytic therapy for ischemic stroke by tPA occasionally results in problems or insufficient therapeutic effects. The results indicated that if tPA was given within the time latency window of up to 3 h it could significantly increase the propensity for cell survival. tPA’s ability to influence different cellular pathways suggest that targeting the desired ones could increase the therapeutic window of tPA in stroke recovery. To provide even better neuroprotection following an acute cerebral infarct, future therapeutics could focus on preventing the neurotoxic damage caused by tPA. In this review, we will discuss the current overview abroad tPA and the current knowledge concerning the natural history of tPA and aim to identify the relevant cellular signaling mechanisms underlying the tPA-mediated effects in-vitro. We also reviewed the present applications of several nanocarriers intended for the administration of tPA in ischemic strokes while also reviewing the biology, thrombolytic mechanism, and pleiotropic roles of tPA in the brain. We’ve also discussed the difficulties and the probable future of tPA-based Nano thrombolysis in stroke treatments.

## Introduction

Ischemic stroke is the second leading cause of death worldwide. However, therapeutic options remain limited (Bao et al. 2022), and tissue plasminogen activator (tPA) is currently the only FDA-approved drug for patients with acute ischemic stroke (Taussky And Couldwell 2022). Studies have demonstrated that treatment with tPA in patients with intracranial aneurysms markedly increases the risk of complications and negatively affects treatment outcomes. Moreover, patients undergoing novel interventions for cerebrovascular disorders, such as aneurysms and AVMs, represent an additional challenge, which further restricts the therapeutic window of this drug (Chen et al. 2025; Tabibkhooei et al. 2025).

Therefore, a comprehensive understanding of tPA’s structure and its effects on neural cells is essential for developing novel strategies to overcome its limitations. Although many studies have been conducted, the need for deeper insights remains critical. Due to the narrow therapeutic window, researchers have explored several approaches to extend it, and targeting strategies are among the most actively investigated. Nanotechnology, in particular, has shown considerable promise in this regard.

In addition, recognizing the neurotoxic side effects of tPA in both intracellular and extracellular pathways may help reduce these adverse outcomes. In this study, we review the structure of tPA, its mechanism of action within the neurovascular unit, and current targeting methods, providing a comprehensive reference in this field. We also include diagrams of chemical structures and schematic representations of nanocarriers for tPA delivery. Further research focusing on faster drug delivery and more specific receptor engagement may help address current knowledge gaps.

## tPA Structure

**Fig. 1:**
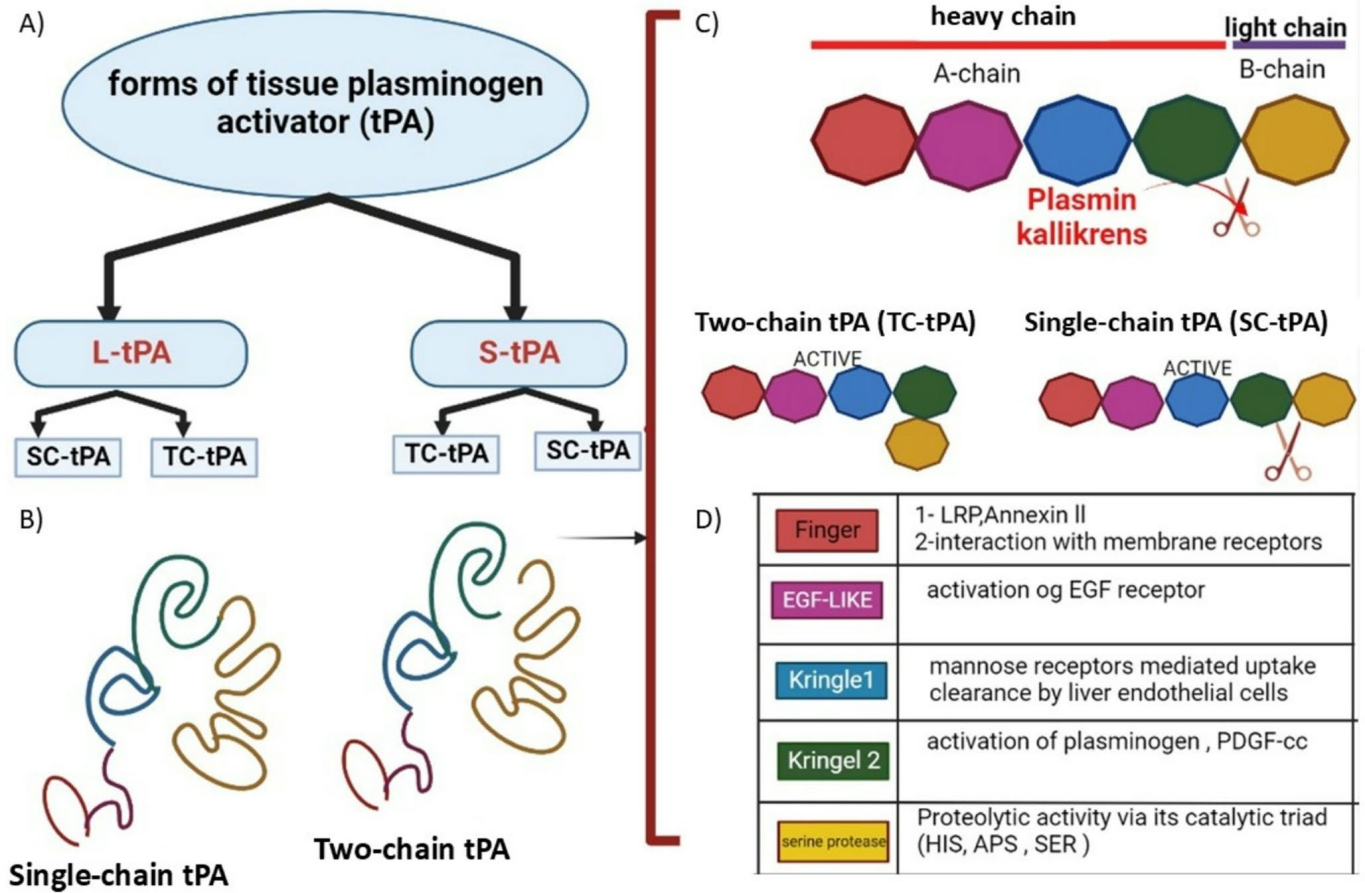
Structural variants of tissue plasminogen activator (tPA), including the short (S-tPA) and long (L-tPA) forms. Each variant exists in both single-chain (SC-tPA) and two-chain (TC-tPA) structures. (**A**) The primary structure of tPA, consisting of four domains shown in different colors. (**B**) The heavy (A-chain) and light (B-chain) segments of tPA, including the finger, EGF-like, kringle-1 and kringle-2, and serine protease domains. Plasmin or kallikrein can cleave tPA at the junction between the light and heavy chains. (**C**) Summary of the functions of each domain. (**D**) Schematic overview of domain roles

There are several forms of tPA (Fig. 1.A). The mature form of tPA is a glycoprotein composed of either 527 or 520 amino acids. Structurally, tPA contains five distinct modules, and its single-chain form can be cleaved by plasmin into a two-chain form (tc-tPA), consisting of a heavy chain and a light chain (Fig. 1.B) (Lin And Hu 2014).The single-chain form is organized into five domains: a finger domain, an epidermal growth factor (EGF) domain, two kringle domains, and a protease domain (Lin And Hu 2014). In tc-tPA, the heavy chain contains the finger, EGF, and kringle domains, while the light chain consists solely of the protease domain (Fig. 1.C). The active site of tPA includes Histidine 322, Aspartic acid 371, and Serine 478 (Wang et al. 2024). Mutations in Serine 478 cause inactivation of tPA and have been shown to play a critical role in its protease-independent functions (Lin And Hu 2014; Olson et al. 2001). Each domain of tPA contributes to the molecule’s pleiotropic functions (Fig. 1.D) (Yepes et al. 2009).The finger domain mediates fibrin binding and is essential for enhancing fibrinolytic activity at lower concentrations (Collen et al. 1988; Larsen et al. 1988). In the brain, additional functions have been attributed to this domain, including involvement in annexin-II and microglial activation (Nicole et al. 2001), crossing of the blood–brain barrier (Benchenane et al. 2005), astrocytic clearance (Cassé et al. 2012), and participation in various signaling pathways (Siao and Tsirka 2002; Pineda et al. 2012). The EGF-like domain shows affinity for EGF and has been linked to both trophic and mitogenic functions of tPA (Liot et al. 2006; Ortiz-Zapater et al. 2007). It is also involved in hepatic recapture of tPA (Hajjar And Reynolds 1994). Additional roles of the EGF-like domain include interactions with platelets and stabilization of the catalytic active site (Yepes et al. 2009).

The kringle domains are stabilized by three disulfide bridges that maintain their looped structures. The K1 domain plays a critical role in tPA uptake by mannose receptors on hepatic endothelial cells in vitro and in vivo, largely due to high-mannose-type glycosylation at Asn117 (KUIPER et al. 1996). The K2 domain, particularly through its lysine-binding site (LBS), is responsible for interactions with and activation of substrates and receptors such as plasminogen, PDGF-CC (platelet-derived growth factor-CC) (Fredriksson et al. 2004), and NMDAR (N-methyl-D-aspartate receptor) (Lopez-Atalaya et al. 2008). Unlike K2, the K1 domain lacks a lysine-binding site (Kim et al. 2003). The catalytic activity of tPA relies on its catalytic triad—His322, Asp371, and Ser478—in which the aspartic acid residue (Asp371) is hydrogen-bonded to histidine (His322), which in turn is hydrogen-bonded to serine (Ser478). This triad is formed within the C-terminal protease domain, enabling enzymatic activity.

### Forms of tPA Structure: long and Short Variants

tPA is initially synthesized as a protein consisting of 562 amino acids. Before being stored in vesicles and subsequently released, a signal peptide of 22 amino acids and a propeptide of 10 amino acids are removed. At the N-terminal end of the molecule, three additional amino acids (Glycine, Alanine, and Arginine: Gly–Ala–Arg) may be present. Depending on whether this tripeptide is retained or removed, either the long variant (L-tPA, 530 amino acids) or the short variant (S-tPA, 527 amino acids) is released, respectively (Jörnvall et al. 1983; Berg And Grinnell 1991) (Fig. 1).

## Sources of tPA in the Body

### Endogenous vs. Exogenous

**Fig. 2:**
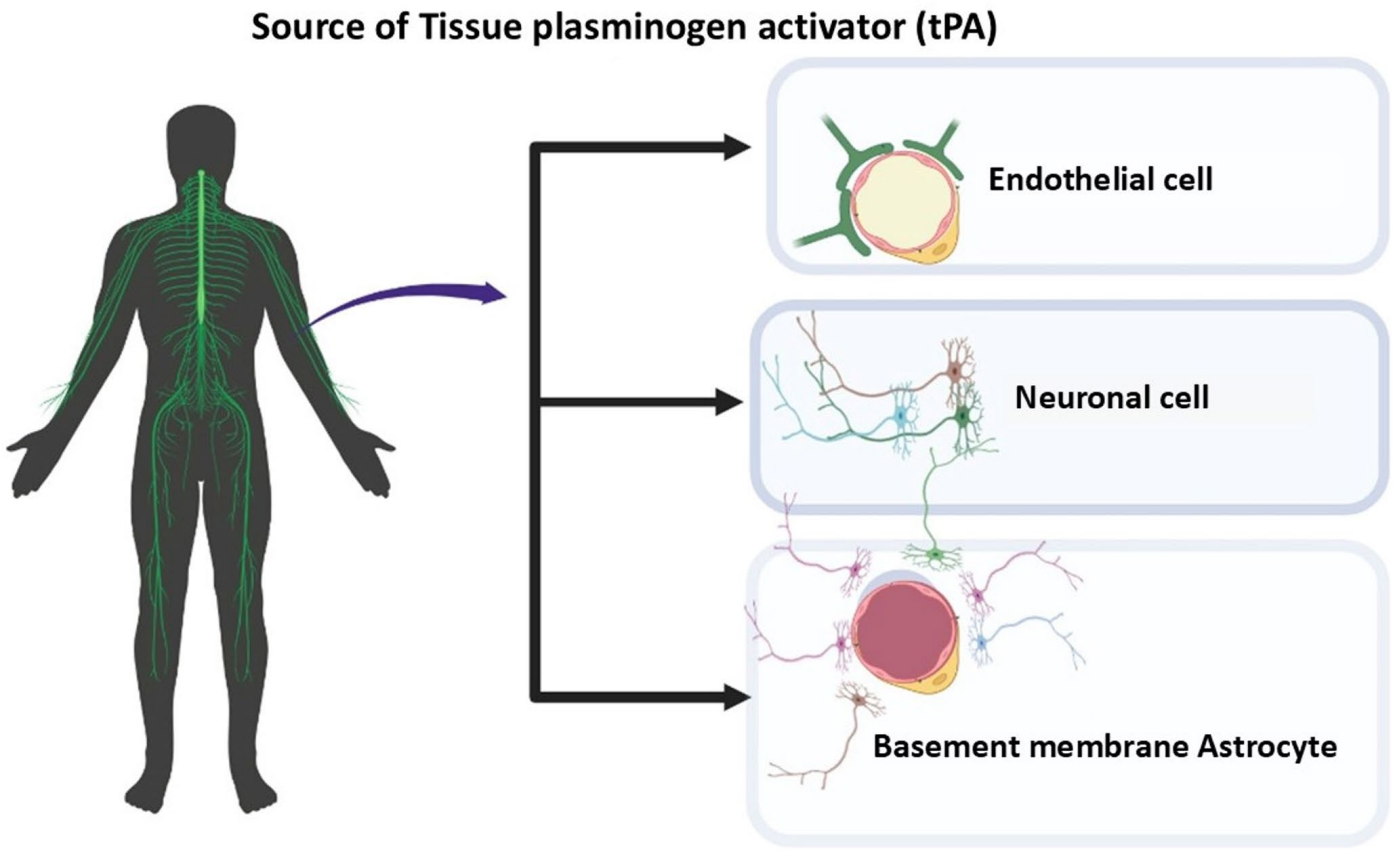
Sources of tissue plasminogen activator (tPA) within the human neurovascular unit

It is important to note that the effects of tPA on neuronal survival may differ depending on whether its origin is endogenous or exogenous. Recent studies suggest that endogenous tPA exerts more neuroprotective effects (Wu et al. 2013; Lemarchand et al. 2016), while exogenous tPA is associated with stronger neurotoxic effects (Parcq et al. 2012). However, exogenous tPA has also been shown to provide neuroprotective effects in hippocampal neurons of tPA-deficient mice (Lemarchand et al. 2016). These findings indicate that factors such as the type of stress paradigm applied and the specific neuronal subtype involved may determine whether tPA (endogenous or exogenous) acts in a neuroprotective or neurotoxic way (Tsirka et al. 1995; Echeverry et al. 2010) (Fig. 2).

### Intracellular Trafficking and Release Mechanisms of tPA

Dendritic tPA granules tend to be less mobile and often reside near postsynaptic spines, positioning tPA for local release at the dendritic (post-synaptic) side of synapses. In contrast, axonal tPA vesicles are more motile (frequently moving retrogradely toward the soma) and supply presynaptic terminals (Lenoir et al. 2019). Consistent with this spatial compartmentalization, significant tPA exocytosis occurs from dendrites (in addition to conventional axonal release), and this secretion is activity-dependent and calcium-triggered. For example, membrane depolarization (high K^+ stimulation) elicits a slow, partial exocytic release of tPA from dendritic spines in vitro, a process that requires extracellular Ca^^2+^ influx. This Ca^^2+^-dependent release mechanism links neuronal activity to tPA’s proteolytic and signaling functions, ensuring tPA is released in an activity-regulated manner rather than constitutively (Lenoir et al. 2019; Lochner et al. 2006).

## Promotion of Cell Survival and Effects on Cellular Signaling Pathways

Neuronal viability depends on complex, interacting networks of signaling pathways that collectively regulate survival, metabolism, and proliferation. Under conditions of cellular stress, whether caused by injury, toxicity, or other insults, the fate of a neuron is largely determined by the input from signal transduction pathways that govern cell survival (Lenoir et al. 2019). Dysregulation within these pathways is often the result of abnormal genetic modifications to critical components or their upstream activators, and such changes can alter signaling in a way that promotes neuronal cell death (Steelman et al. 2011).

At the same time, these pathways are also capable of engaging in pro-survival signaling cascades. In this context, they can enhance proliferation, decrease sensitivity to apoptotic signals, and increase resistance to naturally occurring cell death (Steelman et al. 2011). Several cellular signaling pathways have been identified as key regulators of cell survival. They transmit proliferative signals from membrane-bound receptors to downstream proteins and ultimately to the nucleus, where they control gene expression (Steelman et al. 2011). Among the most important pathways regulating cell growth and survival are the JAK/STAT pathway (Janus kinase/signal transducers and activators of transcription), the ERK pathway (extracellular signal-regulated kinase), the PKA pathway (protein kinase A), and the mTOR pathway (mammalian target of rapamycin), as well as the PI3K/Akt axis. Among these, the PI3K/Akt signaling pathway is of particular relevance to the neuroprotective effects of tPA (Liu et al. 2025).

Akt (also known as protein kinase B) is a serine/threonine kinase that serves as a central downstream effector of PI3K signaling. It plays a pivotal role in promoting cell survival and inhibiting apoptosis, and is also a key regulator of cellular metabolism. In the nervous system, tPA has been shown to activate the Akt pathway. For example, in a cerebral ischemia model, tPA–LRP1 interactions rapidly induced Akt phosphorylation in brain tissue. Likewise, in serum-deprived neuronal cultures, the survival-promoting effect of tPA was abolished by PI3K/Akt pathway inhibition. This suggests that Akt activation may mediate the neuroprotective actions of tPA by enhancing neuronal survival under ischemic conditions (Liu et al. 2025; An et al. 2008; Zheng et al. 2024).

## 4. Specific Function of tPA

tPA, a serine protease, is involved in diverse biological activities, including blood clot breakdown, fibrinolysis, and matrix regulation, primarily through catalyzing the conversion of plasminogen to plasmin (Fredriksson et al. 2004). Because of its thrombolytic properties, tPA is used to treat thrombotic and embolic stroke, but it is contraindicated in hemorrhagic stroke or head trauma (Yepes et al. 2009; Pfeilschifter et al. 2013). Although tPA is recognized as an effective clot-dissolving therapy for ischemia, its clinical application is restricted by a narrow therapeutic window of approximately three hours after stroke symptom onset (Ringleb et al. 2002; Lees et al. 2010). When administered outside this timeframe, tPA is associated with increased neurotoxic effects, which may exacerbate ischemic infarction through activation of several signaling pathways. Additional adverse effects include degradation of the extracellular matrix and increased neurovascular permeability (Yepes et al. 2009). An overview of the putative neurotoxic and neuroprotective effects of tPA is provided below.

### Neuroprotective Effects of tPA

Tissue plasminogen activator (tPA) is widely recognized for its beneficial effects, which are reported to outweigh its drawbacks nearly tenfold (Demaerschalk And Yip 2005). In addition to dissolving blood clots, tPA contributes to post-stroke recovery by preventing neuronal cell death (Lemarchand et al. 2016; Jeanneret and Yepes 2017). During clot formation, tPA is carefully released by endothelial cells and regulated by PAI-1 to maintain vessel patency. Studies have shown that tPA activates neurotrophic proteins such as brain-derived neurotrophic factor (BDNF) and nerve growth factor (NGF), which provide anti-apoptotic support to neurons primarily through the PI3K signaling pathway (Liot et al. 2006; Bruno And Cuello 2006; Barde 2025; Kachuei et al. 2024).

In neural tissue, tPA also promotes axonal growth (Krystosek and Seeds 1981), enhances brain adaptability and synaptic plasticity (Seeds et al. 1995), and improves learning and memory. These effects are independent of its proteolytic activity and are mediated through interactions with receptors such as LDL receptor–related protein 1 (LRP1) and annexin-II (Lee et al. 2007; Kazemi et al. 2024). Furthermore, tPA activates intracellular cascades including Akt, ERK, and JNK, which play critical roles in neuronal protection (Pineda et al. 2012). Experimental studies have demonstrated that both endogenous and recombinant tPA protect neurons from hypoxia and ischemia through NMDA receptor interactions and by upregulating protective molecules such as p21 (Ortiz-Zapater et al. 2007). Additional evidence shows that tPA activates the mTOR pathway, which promotes glucose uptake and enhances neuronal survival under ischemic conditions (Wu et al. 2012). It can also stimulate CREB activity through ERK1/2 signaling, thereby protecting neurons against excitotoxicity (Wu et al. 2013). Moreover, by inducing AMPK activation in astrocytes and endothelial cells, tPA increases lactate production that fuels neurons under metabolic stress (An et al. 2008).

Collectively, these findings identify tPA as a potent modulator of neuronal survival, synaptic remodeling, and energy homeostasis during ischemia and excitotoxic stress. The diversity of these protective effects suggests that tPA acts through multiple receptor-linked mechanisms. The following subsections examine in detail how tPA engages specific receptors including LRP1, annexin-II, and NMDA receptors to activate survival signaling and preserve neurovascular integrity(Yepes 2015).

#### tPA Interaction with LRP1 and Annexin-II Receptors

tPA exerts several of its non-fibrinolytic effects in the brain by binding to cell-surface receptors such as LRP1 and annexin-II. LRP1 is a multi-domain endocytic receptor that can bind tPA and internalize it, thereby regulating extracellular tPA levels. Beyond its clearance role, LRP1 initiates signaling cascades when engaged by tPA; in neurons, tPA–LRP1 interaction transactivates Trk neurotrophin receptors and activates downstream kinases including PI3K/Akt and ERK, promoting neuronal survival and neurite outgrowth. These signaling events occur independently of tPA’s protease activity and contribute to its neurotrophic effects (Herz And Strickland 2001; Mantuano et al. 2013).

In the vascular compartment, however, tPA’s engagement of LRP1 can disrupt neurovascular integrity, binding of tPA to LRP1 on brain endothelial cells triggers PDGF-CC–dependent signaling that increases blood–brain barrier (BBB) permeability. This pathway involves NF-κB, MMP-9, and iNOS activation and contributes to edema and neurovascular injury during ischemia. Annexin-II (annexin A2) is another major tPA-binding protein present on endothelial and immune cells. It acts as a co-receptor that binds both plasminogen and tPA, localizing them to the membrane to accelerate plasmin generation and support vascular patency. However, tPA–annexin-II engagement can also activate microglia and induce pro-inflammatory responses that contribute to neurovascular damage. Thus, through LRP1 and annexin-II, tPA influences receptor-mediated endocytosis, intracellular signaling, and the balance between vascular protection and inflammation (Siao and Tsirka 2002; Herz And Strickland 2001; Mantuano et al. 2013; Kim And Hajjar 2002).

#### Receptor-Coupled Signaling and NMDA Receptor Modulation

Different tPA receptors couple to distinct intracellular signaling pathways, explaining the molecule’s dual neuroprotective and neurotoxic effects. LRP1 generally links tPA to pro-survival signaling; neuronal tPA–LRP1 engagement recruits the PI3K–Akt and MAPK/ERK pathways, promoting cell survival, axonal growth, and plasticity in ischemic models. Conversely, annexin-II–dependent signaling tends to activate mitogenic and stress-responsive pathways; tPA binding to annexin-II, often in cooperation with the epidermal growth factor receptor, triggers ERK1/2 activation and endothelial proliferation through non-proteolytic mechanisms (Mantuano et al. 2013; Macrez et al. 2010).

On glial and immune cells, annexin-II clustering by tPA can activate integrin-linked kinase and NF-κB pathways, leading to inflammation and matrix remodeling. tPA’s interaction with the N-methyl-D-aspartate receptor (NMDAR) represents another distinct signaling route. Early studies showed that tPA binds the GluN1 subunit and potentiates NMDAR activity, increasing Ca²⁺ influx and excitotoxic neuronal death. More recent evidence, however, demonstrates that the two-chain form of tPA (tc-tPA) can activate the MET tyrosine kinase receptor, which promotes internalization and degradation of GluN2B-containing NMDARs. This MET-dependent mechanism suppresses harmful extrasynaptic NMDAR activity and protects neurons from excitotoxicity. Together, these findings indicate that the cellular outcome of tPA signaling, neuroprotection or neurotoxicity depends on receptor selectivity and context. Engagement of LRP1 or MET promotes survival pathways, whereas activation of annexin-II or NMDAR under pathological conditions can amplify inflammation and excitotoxic injury (Macrez et al. 2010; Hedou et al. 2021).

### Neurotoxic Effects of tPA

Although tPA has protective effects, it can also be harmful. Its therapeutic window is very limited, lasting only 3 to 4.5 h after stroke onset. Consequently, only 3 to 8.5% of patients are eligible to receive it, and only 1 to 2% actually benefit (Joshi 2016). Nevertheless, tPA remains the only FDA-approved treatment for acute ischemic stroke. One major concern is its impact on the BBB. In experimental ischemia models, tPA damages the neurovascular unit (NVU), increases barrier permeability, and causes cerebral edema (Abbott et al. 2006; Yang And Rosenberg 2011; Mehrjerdi et al. 2015; Yepes et al. 2003). This damage is linked to the activation of enzymes such as MMPs and PARP, which further impair vascular integrity (Tabibkhooei et al. 2025; Jickling et al. 2014). Similar to VEGFR-mediated signaling, tPA directly influences cerebral vasculature and NVU stability. These processes contribute to vascular permeability and endothelial dysfunction, which share mechanistic features with vascular injury. This relationship has also provided therapeutic insights, as demonstrated in studies on VEGFR tyrosine kinase inhibitors in gliomas and glioblastoma (Habibi et al. 2025). tPA additionally activates PDGF-CC and, through LRP interactions, initiates signaling pathways that worsen NVU disruption (Su et al. 2008; Polavarapu et al. 2007). These mechanisms disturb astrocytic end-feet and activate NF-κB signaling, which increases MMP-9 and iNOS expression, leading to further barrier permeability (Zhang et al. 2007; Scheider et al. 1999; Wang et al. 1998). Evidence from animal models supports these findings: genetic deletion of tPA (Wang et al. 1998), PAI-1 deficiency, or treatment with neuroserpin all reduce ischemic brain damage (Cinelli et al. 2001).

The proteolytic activity of tPA also contributes to extracellular matrix degradation (Chen And Strickland 1997), microglial activation via annexin-II (Yepes et al. 2009), and MCP-1 induction (Sheehan and Tsirka 2005). Interactions between tPA and LRP can further promote ischemic cell death (Polavarapu et al. 2007). Another important mechanism involves NMDA receptors. Studies have shown that excitotoxin-induced neuronal injury occurs in wild-type mice but not in tPA- or plasminogen-deficient mice (Tsirka et al. 1995; Chen And Strickland 1997). Moreover, tPA–NMDA receptor signaling has been implicated in ethanol withdrawal seizures (Pawlak et al. 2005). In summary, although tPA is a critical therapy for ischemic stroke, its dual role as both neuroprotective and neurotoxic highlights the need for continued research. Such work is necessary to balance its therapeutic benefits with its potential risks.

#### tPA-Mediated NMDA Receptor Activation and Neuronal Excitotoxicity

tPA modulates NMDA-type glutamate receptors in a bidirectional, context-dependent manner. Under normal physiological conditions, neurons release small amounts of tPA during activity, which can enhance NMDA receptor signaling to support synaptic plasticity (e.g. during learning and memory). In pathological states such as ischemia or seizures, however, excessive tPA aberrantly cleaves the NR1 subunit of NMDA receptors, thereby amplifying receptor activity and Ca^^2+^ influx. The resulting Ca^^2+^ overload triggers downstream neurotoxic cascades, notably the overactivation of poly (ADP-ribose) polymerase (PARP) due to Ca^^2+^-dependent nitric oxide generation and DNA damage. This PARP hyperactivation depletes cellular energy stores and promotes a caspase-independent cell death pathway (parthanatos) in neurons (Nicole et al. 2001; Jeanneret et al. 2016).

Consistent with this mechanism, studies show that exogenous tPA markedly exacerbates NMDA-induced neuronal death, both in cultured neurons and in vivo. Mice lacking tPA (tPA^–/–) or animals treated with tPA inhibitors (e.g. neuroserpin) exhibit significantly smaller excitotoxic lesions after stroke or NMDA exposure, whereas adding recombinant tPA worsens neuronal loss. These findings illustrate tPA’s dual role: it shifts from a facilitator of normal NMDA receptor signaling under physiological conditions to a potent driver of pathological NMDA overactivation, Ca^^2+^-dependent PARP activation, and excitotoxic neuronal death under stress (Wang et al. 1998; Jeanneret et al. 2016; Fernández-Monreal et al. 2004).

## tPA Controlled Delivery Methodologies

Several tPA delivery strategies have been developed to improve efficacy and safety. Among the most studied approaches are nanothrombolysis, liposomes, ultrasound-triggered thrombolysis, anti-fibrin antibody-targeted tPA, camouflaged tPA, tPA-loaded microcarriers, and nano-modulated delivery systems. Each method aims to optimize thrombolysis while reducing systemic side effects.

### Nanothrombolysis

The primary goal of nanothrombolysis is to prolong the circulation time of tPA in the bloodstream, enabling a reduced overall dosage, precise targeting of thrombus sites, and improved thrombolytic efficacy, while minimizing adverse effects and hemorrhagic complications. Materials used in these delivery systems are designed for low toxicity, high biocompatibility, non-immunogenicity, and biodegradability.

It is important to note that because thrombi are located within systemic circulation, thrombolytic agents only need to reach the occlusive thrombus itself and do not need to penetrate the BBB to reach the brain parenchyma. Instead, novel tPA formulations should be designed to limit unnecessary BBB penetration and to provide adequate protection for brain cells (Prego-Domínguez et al. 2025).

The materials used in nanothrombolysis can be easily surface-modified with functional moieties to improve thrombus targeting (Ren et al. 2024). For passive targeting and enhanced clot penetration, specific formulations such as microbubbles (MBs) and magnetic nanoparticles (MNPs) have frequently been combined with ultrasound or magnetic forces to improve local delivery efficiency (Shakya et al. 2024; Zhang et al. 2025).

### Liposomes

**Fig. 3:**
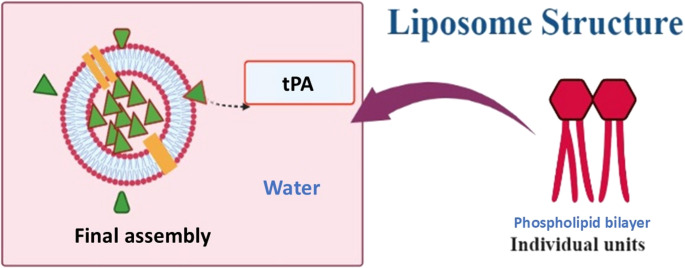
Structure of a liposome encapsulating tPA

Liposomes are widely recognized for their ability to encapsulate therapeutic agents, combined with high biocompatibility and low immunogenicity. By isolating the drug from external conditions, liposomal delivery reduces off-target effects, such as bleeding caused by plasminogen activators (Cai et al. 2022). One limitation of liposomes is their relatively rapid clearance from systemic circulation due to phagocytosis by the reticuloendothelial system (RES), which shortens their half-life. However, studies have demonstrated that encapsulation of tPA into liposomal formulations significantly extends its circulation time. For example, encapsulation into conventional liposomes (EPCL) and PEGylated liposomes (EPC-PEGL) increased the half-life of tPA by approximately 16- and 21-fold, respectively, compared to free tPA. The terminal-phase half-life of free tPA was prolonged from 5.87 min to 50.03 min with EPCL and 132.62 min with EPC-PEGL. Liposomes can also encapsulate gas or fluid to generate echogenic liposomes (ELIP) (Marson et al. 2022). These formulations serve as ultrasound contrast agents and have been employed in ultrasound-assisted thrombolysis. Decoration of liposomes with targeting ligands further enhances platelet binding, thereby extending circulation time (Fig. 3). For example, PEGylated and non-PEGylated liposomes decorated with the fibrinogen gamma-chain peptide sequence (CQQHHLGGAKQAGDV) showed increased affinity for the GPIIb/IIIa receptors expressed on activated platelets. In this study, the half-life of tPA increased from 7 min for free tPA to 103 min for non-PEGylated liposomes and 141 min for PEGylated liposomes (Zhu et al. 2024; Absar et al. 2013).

### Ultrasound-Triggered Thrombolysis

Since the 1970 s, ultrasound has been recognized as a useful tool for thrombolysis. It can be applied either as a standalone therapy or as the trigger component of a drug delivery system for tPA. To maximize thrombolytic activity, contrast agents such as MBs and ELIPs have been extensively investigated. When exposed to ultrasound, these agents enable site-specific drug release and enhance thrombolytic efficacy through cavitation-related mechanisms. This approach, known as ultrasound-based thrombolysis or sonothrombolysis, can also be used as an adjuvant therapy to improve clot dissolution (Brahmandam et al. 2022).

The effectiveness and safety of sonothrombolysis combined with tPA and contrast agents have been evaluated in randomized clinical trials involving stroke patients. An international prospective phase II trial demonstrated that ultrasound enhanced tPA-induced recanalization, although the trend toward improved recovery rates was not statistically significant (Alexandrov 2004). The CLOTBUST-ER trial, the largest phase III study conducted to date, tested a unique operator-independent ultrasound device in patients treated with rtPA after acute ischemic stroke (AIS). The study confirmed the safety of the method but did not show significant improvements in functional outcomes (Rubin and Alexandrov 2022; Alexandrov et al. 2019).

### Microbubbles (MBs) as tPA Carriers

Microbubbles (MBs) are gas- or air-filled microspheres ranging in size from several hundred nanometers to about one micron. They can be used alone or loaded with tPA to promote thrombolysis (Hua et al. 2010; Yan et al. 2017). In vitro and in vivo studies have shown that lyophilized MBs carrying tPA and the Arg-Gly-Asp-Ser tetrapeptide achieved enhanced thrombolytic efficacy with lower tPA doses and potentially reduced hemorrhagic risk (Hua et al. 2010, 2014). In a rabbit thrombosis model, intravenous administration of tPA-loaded MBs followed by ultrasound exposure resulted in complete recanalization (Uesugi et al. 2010). Further research demonstrated that ultrasound-triggered release from MBs improved uPA-loaded nanogel (nUK) thrombolysis, reduced infarct volume, and yielded better clinical outcomes. The nUK formulation also prolonged circulation time and may have protected the BBB without increasing hemorrhagic risk (Teng et al. 2018). Recently, Correa and colleagues developed sub-micrometric CaCO3-templated polymer capsules for rtPA delivery under ultrasound control. Encapsulation of rtPA prevented endogenous inactivation without diminishing thrombolytic activity. In vitro, ultrasound stimulation significantly enhanced clot dissolution, while in vivo, encapsulated rtPA in mice displayed a longer half-life and higher activity compared to non-encapsulated rtPA (Liot et al. 2006).

### Microbubbles as Contrast Agents

**Fig. 4:**
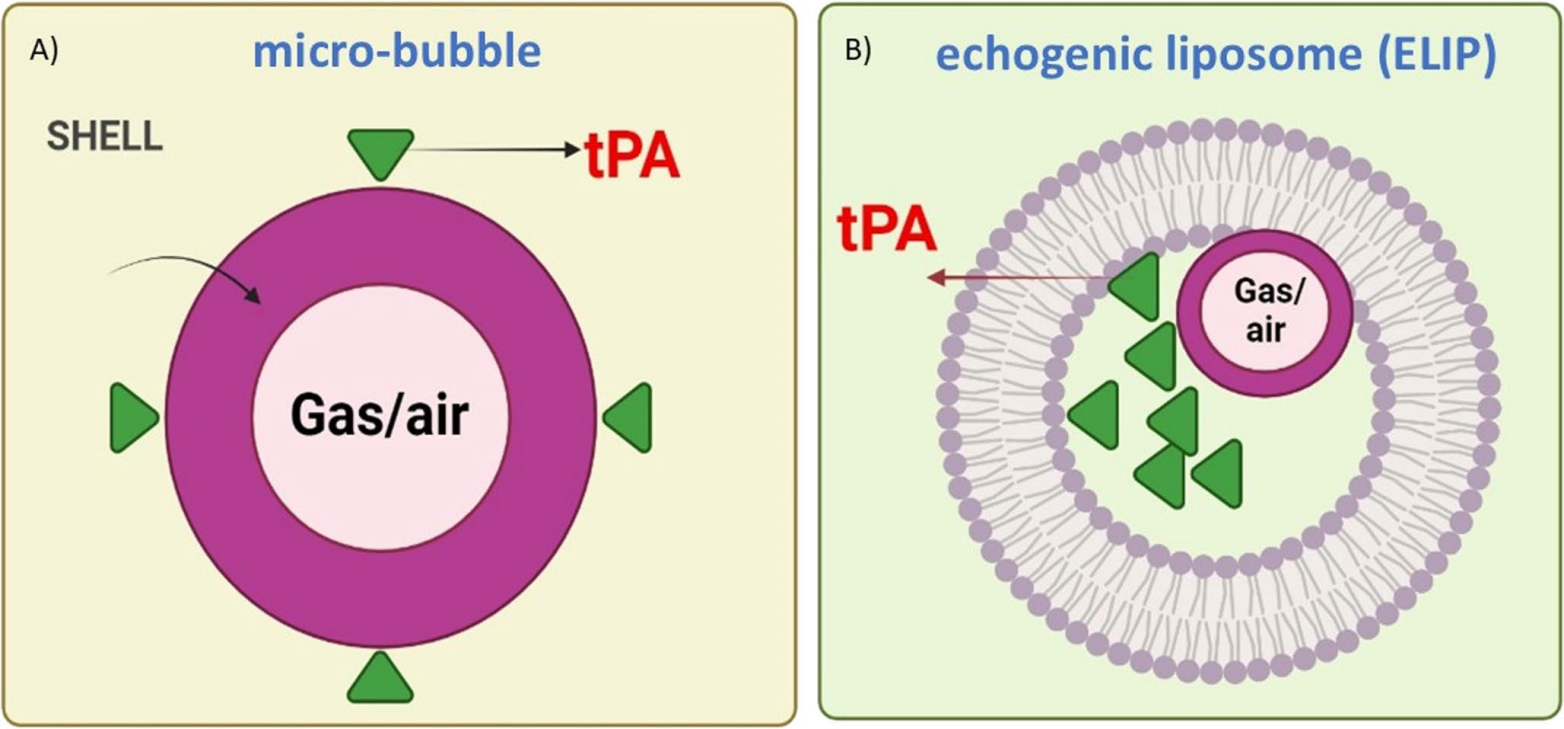
Structure of microbubbles (MBs) and echogenic liposomes (ELIP) used for targeted delivery of tPA

Because of their acoustic properties, MBs were initially introduced as contrast agents for diagnostic imaging (Fig. 4). In ultrasonography, MBs provide higher acoustic impedance than red blood cells, generating stronger echoes and improved image contrast (Hossain et al. 2024). Their ability to act as cavitation nuclei lowers the energy threshold for cavitation and contributes to ultrasound-accelerated thrombolysis (Kazempour And Balogh 2024). Stable cavitation causes MBs to oscillate, generating microstreaming and surface erosion of clots. This mechanical effect enhances clot penetration by fibrinolytic enzymes. In a clinical trial, Molina and colleagues evaluated 111 patients with acute stroke caused by MCA occlusion. Patients were divided into three groups, and complete recanalization was significantly higher in the group that received tPA combined with continuous two-hour transcranial Doppler (TCD) monitoring and galactose-based MBs (Molina et al. 2006).

### Anti-Fibrin Antibody-Targeted tPA

Marsh and colleagues developed a fibrin-specific delivery approach by attaching an anti-fibrin monoclonal antibody to the surface of perfluorocarbon (PFC) nanoparticles. Fluorescence microscopy confirmed the ability of these constructs to target thrombi in a canine model. Because each PFC nanoparticle is capable of carrying up to 40 anti-fibrin antibodies and 400 urokinase molecules, this strategy allows efficient, localized delivery of thrombolytic agents (Marsh et al. 2011). In another study, Wang and colleagues designed an innovative theranostic microbubble (MB) by conjugating anti-GPIIb/IIIa single-chain antibodies (scFvs) with a recombinant thrombolytic agent, single-chain urokinase plasminogen activator (scuPA). This construct enabled both the diagnosis and treatment of thrombosis. The theranostic MBs demonstrated high sensitivity for in vivo thrombus detection, real-time monitoring of thrombus size, and simultaneous thrombolysis in response to the therapeutic payload (Wang et al. 2016; Hanaoka et al. 2022).

### Distribution Strategy Using Camouflaged tPA

Absar and colleagues developed a camouflaged-tPA delivery method tested in multiple experiments. In this approach, tPA was conjugated with low-molecular-weight heparin (LMWH), followed by coating with an albumin-protamine complex (Absar et al. 2013). Co-administration of heparin and tPA resulted in 71% clot lysis, compared with 52% for tPA alone. However, this combination increased bleeding risk, as reflected by a twofold prolongation of activated partial thromboplastin time (aPTT). In contrast, albumin-camouflaged heparin-tPA produced comparable clot lysis activity (70%) to the tPA-heparin combination, but without prolongation of aPTT after one hour of treatment. These results demonstrate the potential of camouflaged-tPA to maintain thrombolytic activity while reducing bleeding risk. Furthermore, tPA-loaded microcarriers may provide targeted thrombolysis with improved safety (Zhu et al. 2024; Binning et al. 2021).

### Delivery Methods Using Microcarriers

Microcarriers (MCs) offer several advantages, including large drug-loading capacity and the ability to co-encapsulate magnetic nanoparticles for improved control of microsphere movement against blood flow (Huang et al. 2019a, b). Torno and Kaminski compared thrombolytic effects of free tPA, tPA mixed with magnetic microcarriers (MMC), and tPA combined with MMC under an external magnetic field (MF) (Spanò et al. 2025). They also evaluated the effects of MMCs exposed to both MF and ultrasound in combination with tPA. Results showed that thrombolysis efficiency improved by 1.7- and 2.7-fold for red and white clots under static, no-flow conditions. A novel magnetic-targeting delivery system was later developed by encapsulating tPA into magnetic poly(lactic acid)-poly(ethylene glycol) (PLA-PEG) microcarriers. This formulation further enhanced the overall thrombolytic activity of tPA (Spanò et al. 2025).

### Targeting with a Magnetic Field

**Fig. 5:**
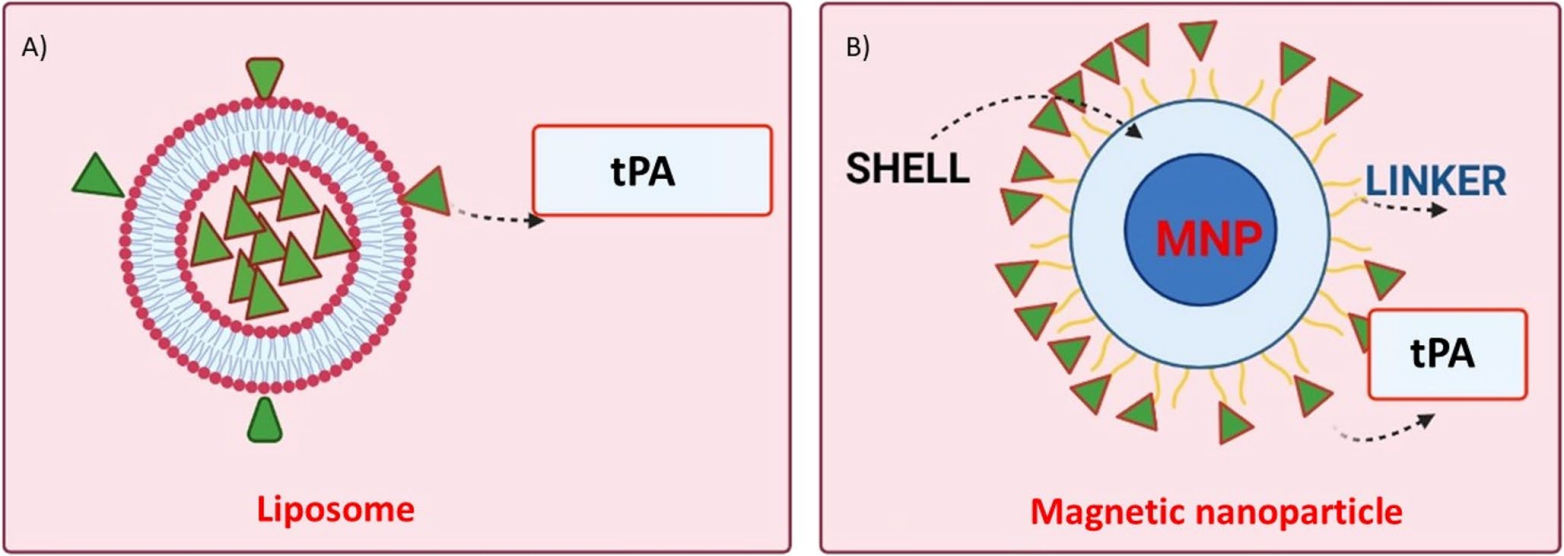
Schematic representation of tPA delivery systems. (**A**) Liposome encapsulating or surface-loading tPA (**B**) Magnetic nanoparticle (MNP) conjugated with tPA through linker molecules, forming a shell around the magnetic core for magnetically guided targeting under an external magnetic field

Magnetic-guided targeting is another promising physical strategy, in which an external magnetic field directs drug-loaded magnetic nanoparticles (MNPs) to specific sites (Saint Victor et al. 2019; Tadayon et al. 2016; Zhang et al. 2021) (Fig. 5). Typically, these MNPs consist of an iron oxide core (Fe3O4 or γ-Fe2O3) coated with organic or inorganic layers. Ma and colleagues generated polyacrylic acid (PAA)-coated MNPs with covalently bound rtPA and demonstrated that intra-arterial delivery restored iliac blood flow to 82% of pre-occlusion levels. This targeted thrombolysis was achieved with only 20% of the standard rtPA dose (Ma et al. 2009). Fe3O4 MNPs coated with poly [aniline-co-N-(1-one-butyric acid) aniline] and conjugated with rtPA also improved thrombolysis efficacy. In vitro testing showed shorter clot lysis times, reduced from 39.2 ± 3.2 min to 10.8 ± 4.2 min, using this formulation. Importantly, restoration of blood flow was achieved without hematological toxicity, again with only 20% of the typical rtPA dose (Chen et al. 2012). Chen and colleagues reported that chitosan-based MNP nanocomposites achieved comparable thrombolysis results with similarly reduced rtPA dosages (Chen et al. 2011). More recently, Huang and colleagues demonstrated the utility of PAA-MNPs as effective carriers for targeted tPA delivery under a rotating magnetic field in a rat model of distal middle cerebral artery occlusion. Their results showed improved thrombolysis efficiency and a reduced infarct size (Ma et al. 2021; Huang et al. 2019a, b).

## Conclusion

The use of exogenous tPA within its therapeutic window remains a valuable treatment option for patients with ischemic stroke, even though the effects of endogenous tPA on neurons are still debated. Further experimental studies are needed to clarify the neuroprotective and neurotoxic effects of tPA.

The pro-survival actions of tPA include activation of EGF receptor signaling, enzyme-II signaling pathways, PI3K, AMPK, and the mTOR–HIF-1α axis. Importantly, these effects occur independently of tPA’s proteolytic activity. In contrast, its neurotoxic effects in adults appear to be closely linked to proteolytic activity through interactions with plasminogen, NMDARs, extracellular matrix components, and inflammatory mediators. At present, clinical data alone are insufficient to determine whether tPA acts predominantly as a neurotrophic or neurotoxic factor, highlighting the need for more research.

To address this issue, it is important to consider the existence of different isoforms of tPA (type I sc-tPA, type I tc-tPA, type II sc-tPA, and type II tc-tPA), the varying affinities of tPA for distinct receptors, and the fact that receptor expression may differ depending on the maturity and regional localization of cortical neurons, particularly those in the hippocampus. Expanding our knowledge of these mechanisms may help to extend the therapeutic window of tPA, thereby improving its clinical effectiveness. The present findings provide a basis for understanding the protective effects of tPA in primary neurons and shed light on the mechanisms that differentiate it from other therapeutic approaches in stroke management.

## Prospective Future

Nanomedicine represents a promising frontier for improving tPA delivery and targeting. Novel strategies involving nanocarriers may enhance the precision of tPA delivery, prolong its circulation time, and reduce the risk of bleeding. These advances hold the potential to optimize the clinical utility of tPA and expand its safe use in the treatment of ischemic stroke.

## Data Availability

No datasets were generated or analysed during the current study.
